# Cost analyses of a web-based behavioral intervention to enhance fruit and vegetable consumption

**DOI:** 10.1186/1479-5868-6-92

**Published:** 2009-12-29

**Authors:** Anna Sukhanova, Debra P Ritzwoller, Gwen Alexander, Josephine H Calvi, Carola Carlier, Jennifer B McClure, Sharon Rolnick, Christine Johnson

**Affiliations:** 1 Institute for Health Research, Kaiser Permanente Colorado, Denver, CO, USA; 2 Henry Ford Health System and Hospital, Detroit, MI, USA; 3 Center for Health Research, Kaiser Permanente Georgia, Atlanta, GA, USA; 4 University of Michigan, Ann Arbor, MI, USA; 5 Group Health Research Institute, Seattle, WA, USA; 6 HealthPartners Research Foundation, Minneapolis, MN, USA

## Abstract

**Background:**

The purpose of this paper is to evaluate costs associated with the online intervention trial, Making Effective Nutritional Choices for Cancer Prevention (MENU), and to connect the findings to the study outcomes.

**Methods:**

Using prospective data collected during the MENU development and implementation phases, we estimated overall costs per person, incremental costs for the three arms of the MENU intervention, and incremental costs per change in fruit and vegetable (F&V) consumption across the studied population. The MENU study was conducted in five HMO sites of the Cancer Research Network. The number of eligible study participants who were enrolled in the study was 2,540. Recruited participants were randomized into (1) an untailored website program, (2) tailored website program, or (3) tailored web program plus personalized counseling (HOBI) via email. The primary measures for these analyses include the total intervention costs, average cost per participant, and the average cost per mean change in daily intake of F&V, stratified by study arm.

**Results:**

The mean change in F&V consumption was greater in both the tailored arm and statistically higher in the HOBI arm relative to the untailored arm. The untailored arm achieved +2.34 servings increase vs. the tailored website arm (+2.68) and the HOBI arm (+2.80) servings increase. Total intervention costs for MENU participants who completed the 12-month follow-up assessment, by study arm, were estimated to be $197,197 or $110 respectively. This translates to $69 per participant in the untailored web site intervention, $81 per participant in the tailored website intervention, and $184 per participant in the HOBI intervention and a cost per average change in F&V consumption to be $35, $27 and $61 respectively.

**Conclusions:**

Providing personalized "tailored" messages and additional personalized support via email generated an additional $12-$115 per participant, over the untailored web program. Incremental increases in F&V consumption associated with the email support arm were associated with considerable increases in intervention costs, suggesting that the most cost effective arm of the MENU study by servings gained was the tailored website.

## Background

Eating patterns lead to a variety of health problems that plague millions of Americans. Research findings confirm a connection between the quality of food consumed and several chronic diseases such as some cancers, coronary heart disease, diabetes, stroke, obesity and osteoporosis [1,2]. In an effort to provide the general public with more information on healthy nutrition, dietary guidelines emerged in the late 1970s that continue to be developed and re-evaluated today. Most nutritional guidelines promote reduced consumption of saturated fat, cholesterol, sodium, and sugar, and increased consumption of fiber-rich foods [3]. Greater need to evaluate public awareness of health risks associated with unbalanced nutrition has coincided with emergence of behavioral interventions targeting nutrition and dietary patterns [4].

Despite increased popularity and importance of behavioral interventions directed towards weight loss and nutrition, very little research has been focused on the cost-effectiveness of such programs. Most research efforts in this area have been directed toward addressing general economic trends and their connection to dietary and lifestyle choices of different socioeconomic groups across the United States [5]. However, limited attention has been directed toward actual program evaluation. Cost analyses can provide essential information related to the magnitude of costs of the intervention relative to the health outcomes achieved, which may further help to determine the best value for program components and facilitate resource allocation decisions.

Numerous programs endorse healthy lifestyle choices with specific emphasis on nutrition (e.g., 5Aday.gov). Personal sessions with dieticians and medical specialists have proven to be effective in achieving significant outcomes and subsequent health benefits for the participants [6,7]. However, frequent personal sessions might not be feasible for people with busy schedules, low insurance coverage, or limited access to health care providers. On the other hand, self-help booklets, guides, and pamphlets, while easy to administer, have not shown beneficial effect on patient outcomes [8]. This suggests that there is a strong need for innovative, affordable, and effective behavioral interventions that can address these limitations [9].

This paper examines intervention and recruitment costs of three versions of an online dietary intervention program assessed in the Making Effective Nutritional Choices for Cancer Prevention (MENU) study. MENU is an Internet-based dietary change randomized intervention program that was designed to promote greater consumption of fruit and vegetables (F&Vs) among members of five geographically diverse integrated health plans [10]. The purpose of this study was to compare the intervention costs and cost-effectiveness of two of tailored behavioral arms of the MENU randomized clinical trial as compared to the untailored arm of the MENU randomized clinical trial [10].

## Methods

### Design

The MENU study was one of the core projects associated with the National Cancer Institute funded HMO Cancer Research Network (CRN) http://crn.cancer.gov/. The CRN consists of the research programs, enrollee populations, and databases of 12 health care organizations in the HMO Research Network at the time of the study [11]. The MENU study was conducted in five CRN sites including the Henry Ford Health System/Health Alliance Plan in Michigan; HealthPartners Research Foundation in Minnesota; Kaiser Permanente Colorado region; Kaiser Permanente Center For Health Research, Georgia; and Group Health Cooperative in Washington state. University of Michigan's Center for Health Communications Research (UM-CHCR) developed and maintained the online intervention programs. This study was reviewed and approved by each site's Institutional Review Board.

As described in detail in Alexander et al [10], the content of tailored arms of the MENU intervention was based on principles from Social Cognitive Theory, the Transtheoretical Model, and the Health Belief Model [12,13]. The intervention phase was designed to last four months from the point of enrolment and participants continued to have website access through the end of the study. All study arms presented web-based educational sessions with the same layout, design, illustrations, and incremental appearance of short optional special features presenting general nutrition and food selection information. Each arm of the MENU intervention was divided into four sessions offered at 1, 3, 13, and 15 weeks post-enrollment. Each session included several web pages of core content, illustrations, optional links to access more detailed explanations. Included in these special features were illustrations of relative serving sizes, nutrition information, and over 300 FV-based recipes. All web-based program components were accessible to subjects through the 12-month study period [9].

The first study arm gave participants access to general information about F&V consumption without offering personalized communications; we refer to this arm as the untailored website intervention. The second study arm, the tailored website intervention, offered tailored information with personalized messages that addressed specific areas for improvement within the participant's behavior profile assessed in the baseline survey. The third study arm provided the same tailored web program with additional counselling, the human online behavioral interaction (HOBI) via e-mail that supported participant-generated strategies, action plans, reminders, and support for the participants. Throughout the paper, we refer to this arm as the HOBI intervention [14].

Follow-up data collection was completed online, with back-up collection by phone as necessary. For the HOBI arm, email interactions were distributed in four individual sessions to coincide with and in response to the four informational sessions over a four months period. Further details associated with the design and implementation of the MENU study have been described elsewhere [10].

### Sample

Participants were recruited from the membership of each health plan, with approximately equal enrolment (n = 500) targeted at each site. Recruitment began in September 2005 and concluded in February 2006 with 2,540 participants enrolling. Eligible participants were between the ages of 21 and 65, were current members of the participating HMO, had no health conditions that prohibited them from implementing the recommended dietary changes, and had internet access and a working email account. The MENU team employed a low labor-intensity recruitment approach [15]. Potential participants were identified via health plan records, mailed a study invitation letter, and invited to visit the study website where they completed a screening survey for eligibility, provided informed consent, and enrolled online. Recruitment mailings were managed locally at each site. Participant questions were triaged centrally, but handled locally as necessary. To boost participation rates, the invitation letter included a $2 bill as an unconditional incentive to visit the website. Enrolled participants received an additional $20 for the completion of each of the three follow-up surveys at 3, 6, and 12 months post-enrollment, as described in Alexander et al [10]. The incentives were given for each completed surveys and were not reflective of reported F&Vs consumption. Thus, we have no reason to believe that incentives influenced likelihood of positive report on F&Vs consumption.

A total of 2540 subjects were included in the analysis of the MENU clinical trial as noted in Stopponi et al [15]. Out of 2540 participants, 847 were randomized into the untailored website intervention arm, 848 participants were assigned to the tailored website intervention arm, and remaining 845 participants were randomized into the HOBI arm [15]. The mean age at baseline was 46.3 years (SD 10.8). Out of 2540 enrolled participants, 1,645 or 65% were female and 895 or 35% were identified as male. 566 (23%) participants were African American and 1,935 (77%) were identified as White or Other. Out of 2540 participants, 192 (8%) identified themselves as Hispanic. Distribution by gender, race/ethnicity, and living status of married or with a partner did not vary across intervention arms. Approximately half of the subjects included in the analysis had some post high school education without a college degree, and half had either a college or post college degree.

### Measures

The primary MENU study outcome was mean change in daily intake of F&V. Baseline, 3-, 6-, and 12-month surveys were designed to capture risk factors, readiness to change, and overall satisfaction with the intervention. F&V consumption was measured by the NCI F&V All Day Screener, not including mixed vegetables dishes or fried potatoes, resulting in a 16-item questionnaire consisting of detailed questions on portion sizes and specified food consumption [15].

Validity assessments of the NCI fruit and vegetable screener have shown that this instrument performs well in comparison to the gold standard measure of multiple 24-hour dietary recalls [16]. Recent analysis of the NCI fruit and vegetable screener showed that participants tend to over-estimate daily servings using the NCI FV screener compared to the 24-hour dietary recall and correlations between these two measures are higher for women and lower for men [17].

The primary measures in this analysis of the MENU program are the intervention cost components and marginal cost of additional serving of F&V that resulted individually from the untailored, tailored, and HOBI arms of the MENU study.

### Cost analysis procedures

Consistent with our previous work, to accurately calculate implementation and recruitment costs, we created a detailed accounting system that captured all implementation and recruitment attributable expenses [10,18]. Although for some projects assessment of research and development costs might be of utmost importance in the context of future dissemination, given the scope and budget limitations of the MENU project, our efforts were directed solely towards intervention related costs. In addition, we made considerable efforts to track recruitment costs, the importance of which is often overlooked by many programs. In most research projects, labor input becomes a major component of intervention and recruiting efforts; therefore we paid extra attention to documenting labor activities directed toward these phases of the project.

To better track study staff efforts and associated costs, we created tailored cost-capture templates that were based on study staff responsibilities at each site [10]. Each template consisted of relevant task categories representing each person's involvement in the project based on their job category. For example, project managers' templates were the most intricate, reflecting the range and intensity of their coordination and patient tracking effort on the project, while templates for research associates doing mailings were less so.

For consistency in interpretation, tasks were captured in hours spent by each individual contributor. To better understand project progression, data from templates were collected and analyzed on a monthly basis, and recorded hours were transferred into the corresponding dollar amounts based on the salary for that job category. To establish consistency in wage rates across different sites, we utilized data from the Bureau of Labor Statistics (BLS) http://www.bls.gov/. Under a one-site scenario we would use real wages and apply BLS estimates in the sensitivity analyses. However, for the purposes of the MENU project, BLS wage estimates were used in order to avoid inconsistencies and wage bias.

In addition to the labor component, we accounted for other intervention-related expenses. Each individual report included an expense section for recording supplies, printed materials, and other project-related expenses. Finally, to further enhance our knowledge of every-day project activities, we scheduled personal interviews, attended staff meetings, conference calls, and maintained close contact with key personnel.

After the data had been collected and assigned the appropriate dollar values, we created one report that combined all task categories and a complete intervention timeline of the MENU project. Since our focus was directed solely toward the implementation and recruitment phases, we excluded any tasks and expenses that dealt with intervention development, data collection, or other activities unique to the research process other than intervention delivery. Detailed descriptions of tasks performed and a limited timeline for data collection allowed us to exclude any costs that were not associated with recruitment and implementation.

Furthermore, complete cost information enabled us to calculate incremental costs applied across the three study arms, as well as to differentiate between recruitment and intervention cost categories. By extracting cost associated uniquely with each study arm, we were able to determine the incremental cost per arm per participant. In addition, by separating recruitment and intervention costs, we established recruitment-only incremental cost and intervention-only incremental cost per each arm participant. For purposes of comparison, we also combined intervention and recruitment costs and determined total incremental cost per participant in each of the three study arms.

To calculate the incremental change in main outcome, the mean change in servings, we estimated the cost per participant in each arm and applied these data to estimate the average change in servings of the daily servings of F&V, attributable to each arm of the intervention, to form cost-effectiveness ratios.

### Intervention Inputs

Primary intervention inputs associated with the MENU project consisted of labor and technology components. Intervention staff members did not have any direct contact with study participants, so their main efforts were directed toward tracking the progress of the participants, addressing participants' questions and/or concerns, mailing coordination, and quality data assessment. In addition, bi-monthly conference calls and frequent e-mail correspondence were necessary to ensure effective coordination across the different sites. UM-CHCR was responsible for providing technical support to all sites, monitoring web operation, timely response to the technical issues, and operational assistance to study participants.

### Sensitivity Analysis Related to Intervention Capacity

The completion rate was nearly 80% or greater per survey. Of 2,540 recruited participants, 2016 (79.4%) competed the 12 month survey, with 1,788 (70.4%) providing complete data for assessing change in dietary intake. Study outcomes were analyzed using the entire enrolled sample (i.e., intent-to-treat analysis) verses those who completed the 12 month food intake survey (i.e., respondent analysis).

## Results

As noted in Table 1, and described in detail in Alexander et al [10], across the three arms the total mean servings of F&V, adjusted for baseline intake, increased by more than two servings (p > .001), with increases observed at 3 months and then maintained at the one year follow-up. The mean change was greater in both the tailored arm and statistically higher in the HOBI arm relative to the untailored arm.

**Table 1:** Primary Outcome Changes in Daily Servings of Combined F&V Based on 12 months data

	N	Mean Change	Adjusted Mean Change
Untailored Web	611	+2.24	+2.34
Tailored Web	599	+2.81	+2.68
Tailored Web + HOBI	578	+2.77	+2.80

Table 2 provides a brief overview of the main cost components. The combined recruitment and intervention cost for the MENU project was $250,067. Recruitment and intervention costs make up 21% and 79%, or $52,870 and $197,197, respectively. Recruitment and intervention costs combined translate to an overall average cost of $99 per enrolled MENU participant, or $71 per untailored website participant, $78 per tailored website participant, and $147 per HOBI participant.

**Table 2:** MENU Project Cost Components

Cost Element	Direct	Indirect	Direct + Indirect
***Recruitment***			
Project Managers	$28,350	0	$28,350
Research Assistants	$11,230	0	$11,230
Technical Support	$8,210	0	$8,210
Recruitment Incentives	$5,080	0	$5,080

TOTAL RECRUITMENT	$52,870		$52,870
***Intervention***			
Meetings	$47,285	0	$47,285
Technical Support	$30,360	0	$30,360
HOBI component	$58,052	0	$58,052
Personnel	0	$61,500	$61,500

TOTAL INTERVENTION	$135,697	$61,500	$197,197

**RECRUITMENT + INTERVENTION**	**$188,567**		**$250,067**

Table 3 provides a brief summary of the per participant costs across each of the expense categories. Costs reflect the entire enrolled sample at the baseline relative to the 12-month responders. (e.g., cost notes changes in the denominator) Our analyses indicate that the total intervention costs for these two groups are relatively similar. Due to the fixed nature of major cost components, total intervention cost would remain unchanged despite possible variations in the participatory pool. Thus whether we consider entire enrolled sample or just the 12-month responders cohort, the total intervention cost would remain unchanged, $197,197.

**Table 3:** Incremental Cost Per Change in Combined F&V Daily Servings per Study Participant

	Across Arms (MENU participant)	Untailored Web Arm	Tailored Web Arm	Tailored Web + HOBI Arm
***Recruitment***				
At baseline N = 2,540)	$21	$21	$21	$21
At 12 months (N = 1,788)	$30	$30	$30	$30
				
***Intervention Technical Support***				
At baseline (N = 2,540)	$12	$7	$14	$14
At 12 months (N = 1,788)	$17	$10	$20	$21
				
***Intervention***				
At baseline (N = 2,540)	$78	$50	$57	$126
At 12 months (N = 1,788)	$110	$69	$81	$184
				
***Recruitment + Intervention***				
At baseline (N = 2,540)	$99	$71	$78	$147
At 12 months (N = 1,788)	$140	$98	$110	$215

Due to the low labor intensity, recruitment for the MENU study was not a major cost component and involved minimal efforts from programmers, project managers, and research assistants at each site. The recruitment cost per enrolled participant was $21. The average recruitment cost per 12 months respondent was $30.

Intervention cost per enrolled participant can be summarized as follows: $50 per participant in the untailored website intervention, $57 per participant in the tailored website intervention, and $126 per HOBI participant. Intervention cost per 12 month respondent was $69 per participant in untailored web-based intervention, $81 per participant in tailored website intervention, and $184 per HOBI participant.

Website support was a crucial component of the intervention process. Thus, we paid extra attention to the website-related costs accumulated during the intervention stage of the project. During the intervention phase, $30,360 was spent on website upkeep and operations (Table 2). This translated to $12 per enrolled participant and $17 per 12 month respondent (Table 3). More specifically, data suggest 80% of support efforts were directed to manage the tailored web and HOBI arms, and 20% of technical resources were directed towards the untailored website intervention. This distribution of web-related support costs translated to $7 per enrolled untailored website participant and $10 per 12 month untailored website respondent, $14 per enrolled tailored website participant compared to $20 per 12-month tailored website respondent, $14 per enrolled HOBI participant and $21 per 12-month HOBI respondent (Table 3).

Incorporating the main MENU intervention outcomes (Table 1) with cost analyses summarized in Table 2, the marginal cost per 12-month untailored web arm respondent, 12-month tailored web arm respondent, and 12-month HOBI respondent translates to $35, $27 and $61 per additional fruit and vegetable serving, respectively. Based on our analyses, tailored web arm intervention was the least expensive option per additional fruit or vegetable serving. Overall, an additional $12-$115 per participant, respectively, over the untailored web program, resulted in an increase in F&V consumption, compared to a general or impersonal approach.

## Discussion

We estimated the total costs of enrollment and intervention delivery across study arms in the successful online, multi-site MENU trial. To our knowledge, this paper is the first to describe the actual intervention costs associated with a purely web-based behavioral intervention relying on email reminder prompts. Our hope is that the analysis and findings described here will provide detailed methodology and extensive cost data to inform decision makers who may contemplate dissemination of the MENU program, or a similar online behavioral intervention, and provide reference for future research in this area.

We estimated the total intervention and recruitment costs and dissected intervention cost across each study arm. Our results suggest that the modest total intervention cost per participant who completed the final survey and across all three study arms arm ranged from $69 - $184. Even lower estimates were found when we used intent to treat model and based costs on the entire enrolled sample. However, considering the study's participating sites were limited to HMOs and medically-insured population, our study limitations include possibly significant cost and study design variations if applied to different settings and different populations. Given the scope of the study, it can be argued that population in the MENU study might be significantly different from the US adult population in terms of age, sex, and education levels. Thus conclusions of the study could be difficult to compare with interventions where the total spectrum of population has been exposed.

Considering differences between study arms and subsequently achieved results, the tailored website intervention appears to be the most cost-efficient option. As noted in Table 1, the tailored website arm achieved an average of +2.68 servings increase vs. a +2.80 servings increase in the HOBI arm. The difference in the servings change outcomes is minimal, yet the cost per additional serving is very significant, $61 per additional serving in the HOBI arm and $27 per additional serving in the tailored website arm. From our calculations, it may not be reasonable to invest additional $34 to achieve an improvement of 0.12 serving in fruit and vegetable. We acknowledge that our analyses were based only on the 12-month follow-up results, and we can not estimate any longer term dietary assessments.

Research data indicate that better nutrition can save $71 billion annually spent on medical bills, productivity loss, and premature deaths from conditions caused by poor diet (healthyamericans.org/reports/obesity/ObesityReport.pdf). For instance, studies show that increased consumption of fruits and vegetables has a protective effect against some types of cancer [19]. Thus extensive research efforts have been directed to provide information and support that would assist the public to adopt healthier eating habits and subsequently reduce risk factors for health complications [20,21]. This study demonstrates that for a modest amount of resources, payers such as Medicare or health plans such as those participating in this study could provide web-based programs for their members to aid with dietary improvements.

Widespread internet exposure has encouraged the emergence of numerous behavioral interventions designed to be delivered via the web [22,23]. The internet offers a unique method of behavioral implementation as well as encourages self-managed behavioral change [24-26]. Evidence suggests that many participants respond to tailored information not involving intensive interpersonal communications with health providers [27,28]. Easily accessible, web-based programs prove to be cost efficient as well as effective [29]. Evidence also suggests that study participants achieve greater results when assigned to the tailored vs. information only condition [30]. For instance, a web-based weight management program conducted at Kaiser Permanente with 2862 enrolled participants proved to be more effective among participants assigned to the tailored arm than those assigned to the information only arm. These participants reported greater weight loss than the cohort assigned to the information only arm [12]. The MENU study confirms these findings by demonstrating better outcomes in the cohort randomized to the tailored arm with HOBI. As our study demonstrated, participants in all three arms reported improvements in daily servings; however, HOBI participants increased consumption of fruits and vegetables significantly more than those assigned to the untailored website.

Overall, web-based behavioral interventions have a great potential to serve as a viable alternative that can affect millions of people at a relatively low cost. These types of programs might be especially appealing to the large health care organizations that target large portions of their memberships with disease prevention programs. With new technological innovations, and greater numbers of people having access to the internet and email, the process of program delivery can potentially become further automated and subsequently reduce implementation costs. Sensitivity analyses might be important in determining the cost range of future disseminations. Technological progress as well as alternative discount rates, variation in labor inputs, variation in market wage rates, and change in study outcomes can affect cost fluctuations. Broader research on cost-effectiveness of web-based interventions is necessary in order to perform comparative analyses across various interventions and thus better understand cost-effectiveness of different programs.

## Conclusions

Based on the intervention cost results derived from the MENU project, we can conclude that web-based behavioral interventions can present an inexpensive, flexible, and potentially effective alternative for delivering a behavioral intervention to a broad audience in multiple geographic locations. Furthermore, to achieve significant increase in fruit/vegetable consumption, it might be sufficient to use tailored web intervention without supplemental email counseling. Further research is needed to determine whether similar interventions can be effectively applied across various socioeconomic and ethnic populations at the various dissemination sites with similar cost outcomes.

## Competing interests

The authors declare that they have no competing interests.

## Authors' contributions

All authors contributed equally to this work. All authors read and approved the final manuscript.
